# Particulate Guanylyl Cyclase A/cGMP Signaling Pathway in the Kidney: Physiologic and Therapeutic Indications

**DOI:** 10.3390/ijms19041006

**Published:** 2018-03-27

**Authors:** Yang Chen, John C. Burnett

**Affiliations:** 1 Biochemistry & Molecular Biology Graduate Program, Mayo Graduate School, Rochester, MN 55905, USA; 2 Cardiorenal Research Laboratory, Department of Cardiovascular Medicine, Mayo Clinic, Rochester, MN 55905, USA; burnett.john@mayo.edu

**Keywords:** particulate guanylyl cyclase A, natriuretic peptide, cGMP, renal, protein kinase G, acute kidney injury

## Abstract

The particulate guanylyl cyclase A (pGC-A)/cGMP pathway plays important roles in regulating renal physiological function and as well as in counteracting pathophysiological conditions. Naturally occurring peptide pGC-A activators consist of atrial natriuretic peptide (ANP), b-type NP (BNP), and urodilatin (URO). These activators bind and activate pGC-A, generating the second messenger cyclic 3′,5′ guanosine monophosphate (cGMP). Cyclic GMP binds to downstream pathway effector molecules including protein kinase G (PKG), cGMP-gated ion channels, and phosphodiesterases (PDEs). These mediators result in a variety of physiological actions in the kidney, including diuresis, natriuresis, increased glomerular filtration rate (GFR) and organ protection, thus, opposing renal cellular injury and remodeling. Downstream proteins regulated by PKG include collagen 1 (Col-1), transforming growth factor beta (TGF-β) and apoptosis-related proteins. In addition to their physiological regulatory effects, pGC-A/cGMP signaling is critical for preserving renal homeostasis in different renal diseases such as acute kidney injury (AKI). Regarding therapeutic options, native pGC-A activators have short half-lives and their activity can be further enhanced by advances in innovative peptide engineering. Thus, novel designer peptide pGC-A activators with enhanced renal activity are under development.

## 1. Introduction

**Figure 1:**
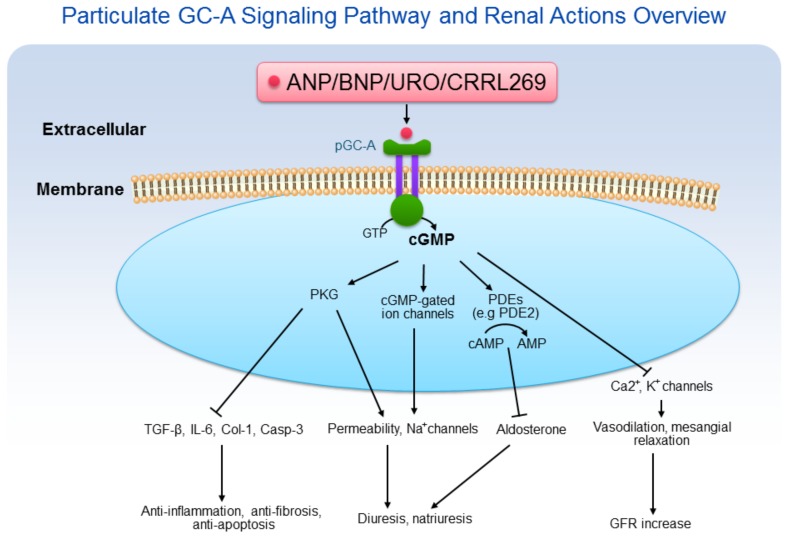
Particulate GC-A signaling pathway and renal actions overview. ANP/BNP/URO/CRRL269 activate pGC-A receptor, generating second messenger cGMP, which binds to protein kinase G (PKG), cGMP-gated ion channels, and phosphodiesterases (PDEs). Cyclic GMP induces pluripotent biological actions, including diuresis, natriuresis, anti-inflammation, anti-fibrosis, anti-apoptosis, and GFR increase.

Peptide-based pGC-A activators and downstream cGMP signaling pathways possess acute and chronic beneficial effects on the kidney. In contrast to soluble guanylyl cyclases (sGC) which are present in the cytosol, pGC-A receptors are membrane bound on the cell surface. Native pGC-A activators in humans include ANP, BNP, and URO. After binding to the extracellular binding domain of pGC-A, part of the intracellular domains of the receptor are activated. The catalytic activity of pGC-A converts GTP to cGMP, thus elevating intracellular cGMP levels [1]. As the second messenger, cGMP mediates a variety of biological actions, including diuresis, natriuresis, enhanced GFR and renal protective processes [2] (Figure 1). Because of the multifaceted renal actions, pGC-A activators have emerged as potential renal protective therapies most importantly for the prevention and treatment of acute kidney injury (AKI) which is a focus of this review.

AKI is a challenging problem for clinicians and a major global burden for patients. AKI is defined by an abrupt decline in GFR which is usually reversible. This results in the elevation of serum creatinine, blood urine nitrogen (BUN), and other metabolic products [3]. AKI has an overall frequency of 6.4% in community acquired and hospital acquired subjects [4] and is especially common in critically ill patients, in whom the prevalence of AKI is greater than 57.3% at admission to the intensive-care unit [5]. Despite its high frequency in critically ill patients, AKI is also a risk factor for chronic kidney disease (CKD), heart failure (HF), and death. Specifically, the risk of developing CKD is higher in patients with AKI compared with those patients without [6]. In a meta-analysis, Coca et al. found patients with AKI present higher risk for CKD (hazard ratio 8.8), end stage renal disease (ESRD, hazard ratio 3.1), and mortality (hazard ratio 2.0). AKI is also associated with acute HF. In a large epidemiological study consisting of 300,868 subjects, AKI was found to be associated with 23% increased risk for incident HF [7]. However, therapeutic interventions targeting AKI are limited. Clinical trials investigating drugs such as *N*-acetylcysteine, sodium bicarbonate or erythropoietin failed to show positive results in AKI patients [8]. Thus, developing novel drugs targeting preservation of renal function and long-term organ protection is of high importance.

Despite failed clinical trials for AKI as discussed above, the use of pGC-A activators has demonstrated efficacy. In two separate human clinical trials, Mentzer et al. [9] and Sezai et al. [10] documented that recombinant ANP and BNP infusion exerted sustained renal benefits in patients undergoing cardiac surgery. These benefits included declines in serum creatinine, hospital stay and mortality. Thus, pGC-A/cGMP has the potential to be an innovative area of drug discovery for AKI. Here in this review we discuss how pGC-A/cGMP pathway regulates renal function and how novel pGC-A activators may provide potential therapeutic options for different renal diseases.

## 2. Particulate GC-A/cGMP in Sodium and Water Homeostasis

Natriuresis and diuresis are important renal actions produced by pGC-A activators. Numerous studies support the potency of pGC-A activators in animals and humans. In rodents [11] and canines [12], pGC-A activators infused as a bolus injection or continuous infusion increased sodium and water excretion. Specifically, in 1984, our laboratory demonstrated that intravenous infusion of ANP significantly increased water and sodium excretion in normal canines compared to baseline [12]. Furthermore, in a clinical trial in normal human subjects [13], ANP infusion resulted in robust natriuretic and diuretic effects, which were consistent with what observed in animal models. Additionally, in HF patients, BNP infusion reduced the use of diuretic drugs compared to standard of care [14]. Thus, pGC-A activators are recognized as potent naturally occurring diuretic and natriuretic agents. The physiological and molecular mechanisms behind the obvious potency observed in vivo are multifactorial and may involve enhanced GFR by both increasing the ultrafiltration coefficient of glomerular membrane, increased glomerular hydrostatic pressure as well as reducing sodium reabsorption at both the proximal tubule and inner medullary collecting duct (IMCD) which are all sites in the nephron in which pGC-A is abundant. Regarding the diuretic effects of pGC-A activators, studies have investigated diuretic actions of ANP in which exogenous cGMP directly reduced vasopression stimulated osmatic water permeability in rat IMCD cells [15]. ANP was also reported to modulate water-channel protein aquaporin-2 (AQP2) translocation during its diuretic actions [16]. In the study by Wang et al., diuresis induced by ANP infusion was not accompanied by changes in subcellular localization of AQP2 in the IMCD, whereas there was a marked increase in apical targeting of AQP2 in IMCD after ANP infusion, indicating a compensatory effect to reduce volume depletion in response to prolonged ANP infusion [16]. These studies suggested that the effect of ANP/cGMP on osmatic water permeability contributed largely to in vivo diuretic actions. Additional studies directly performed fluid absorption experiments in rat proximal tubules, and demonstrated that ANP as well as cGMP significantly attenuated water absorption stimulated by norepinephrine [17].

Notably, fluid excretion accompanies sodium excretion. As the important mediator, cGMP induces diuresis and natriuresis through cGMP-bound ion channel modulation. Cyclic GMP-bound ion channels such as the amiloride-sensitive Na^+^ channel and Na^+^-K^+^-ATPase (NKA) pump regulate renal tubular Na^+^ uptake or excretion. These two ion channels/pumps directly mediate Na^+^ ion in and out of the renal tubules. Cantiello et al. [18] observed that ANP and a cGMP analogue directly inhibited Na^+^ uptake through amiloride-sensitive Na^+^ channel inhibition in swine renal epithelial LLC-PK1 cell line. Consistent with the discovery by Cantiello et al., Light et al. [19] demonstrated ANP and cGMP inhibited amiloride-sensitive Na^+^ channel Na^+^ absorption in renal IMCDs. Also, the pGC-A/cGMP pathway inhibits NKA. ANP, cGMP analogs as well as the non-specific phosphodiesterases (PDEs) inhibitor IBMX were shown to inhibit Na^+^ dependent oxygen consumption (oxygen consumption is a parameter measuring NKA activity) in IMCDs [20,21]. Furthermore, in the study by Scavone et al., the authors performed experiments to show that ANP and cGMP directly inhibit ouabain-sensitive NKA in rat medullary slices with the NKA enzymatic assay [22]. Therefore, pGC-A/cGMP reduces Na^+^ uptake through directly inhibiting Na^+^ channels in the kidney.

In addition to ion channels, investigations have reported that another downstream protein of cGMP, PKG, contributes to the natriuresis of ANP/cGMP [22,23,24]. Light et al. showed that active exogenous PKG directly inhibited the Na^+^ channel in IMCDs. Jin et al. also provided evidences that ANP/cGMP/PKG pathway could stimulate natriuresis directly in renal tubules independent of hemodynamic changes [24]. In another study, investigators discovered that the PKG inhibitor KT-5823 reversed NKA inhibition by ANP, which also suggested PKG is involved in NKA inhibition by pGC-A [22].

Additionally, cGMP binds and activates a member of PDEs, called PDE2. PDE2 has a cGMP binding motif and cGMP directly regulates its activity. PDE2 signaling pathway was reported to reduce aldosterone, an important water/sodium retaining hormone regulator [25,26]. In bovine adrenal glands and zona glomerulosa cells, ANP activates PDE2 and reduces aldosterone levels generated from adrenal cells and tissues [25,26]. In addition, evidence from animal studies and a human clinical trial clearly supports pGC-A activation in the inhibition of aldosterone levels in vivo [27,28]. Thus pGC-A/cGMP/PDE2 signaling is another pathway involved in promoting water/sodium excretion. PDE5 is another cGMP-binding PDE, which is abundantly expressed in the kidney and it degrades cGMP. It is suggested that PDE5 contributes to modulation of natriuresis through the degradation of cGMP. To prove the hypothesis, Chen et al. administered PDE5 specific inhibitor in heart failure canines and demonstrated that PDE5 inhibition elevated cGMP and more importantly increased natriuresis [29]. Hence PDE5 is a negative regulator for cGMP mediated natriuresis.

## 3. Particulate GC-A/cGMP Mediates Glomerular Function

GFR is a critical parameter characterizing normal kidney function. As described above, a decline of GFR is a cardinal characteristic in AKI patients. In animal studies, GFR is usually calculated by inulin clearance method. After continuous infusion of inulin at a fixed concentration, inulin clearance is calculated as: urine volume rate x urinary inulin concentration/ plasma inulin concentration. In the clinic, estimated GFR (eGFR) is calculated based on measured serum creatinine, with adjustment of age, sex and ethnicity. Renal cGMP, an important downstream messenger of guanylyl cyclase, has been suggested to contribute to GFR maintenance and its increase [12,30,31,32]. Published studies documented the increase of GFR by pGC-A activators in different species, including rodents [30], canines [12], and humans [31]. Specifically, in the study by Huang et al., they successfully demonstrated that a cGMP analog dose-dependently increased GFR in rats, mimicking the GFR enhancement effects observed with ANP [30]. Another study conducted in our laboratory also clearly supported the role of cGMP mediated GFR increase in canines and canine glomeruli. In this study, the designer dual guanylyl cyclase activator cenderitide elevated GFR and the increase was accompanied by a markedly increased in glomerular cGMP levels [33]. Furthermore, an elegant study using the pGC antagonist HS-142-1 clearly supports the role of pGC-A/cGMP in the regulation of GFR [34]. Specifically, HS-142-1 administered intravenously significantly attenuated GFR in diabetic rats compared to a control group without HS-142-1 injection [34].

Further studies of mechanisms of pGC-A/cGMP mediated increases in GFR demonstrate that vascular smooth muscle cell (VSMC) relaxation, changes in renal arterial perfusion pressure as well as reduction of mesangial cells contraction play a role. Importantly, pGC-A activators are known to relax renal vessels [2]. Vasorelaxation by cGMP is mediated by decreases in intracellular Ca^2+^ levels and K^+^ channel inhibition. Specifically, work by Rashatwar et al. reported that the cGMP analog 8-Br-cGMP abolished KCl mediated VSMC Ca^2+^ increases and exogenous PKG increased Ca^2+^ ATPase activity, which contributes to a Ca^2+^ decrease within the cells [35]. Furthermore, ATP-sensitive K^+^ channels contribute to vascular tone and ANP as well as 8-Br-cGMP were reported to inhibit ATP-sensitive K^+^ channels present in the VSMC [36]. Renal vascular events are involved in GFR modulation. In our laboratory, we previously proved that reduction in renal artery perfusion pressure of renal blood flow autoregulation by suprarenal aortic clamping prevented the GFR increase observed during ANP infusion [37]. Furthermore, in the rat glomeruli, pGC-A/cGMP induces afferent arteriolar dilatation and efferent arteriolar constriction, generating a rise in the glomerular capillary hydraulic pressure and thus an increase GFR [38,39]. Evidence of the cGMP pathway stimulating afferent arteriolar dilation is also supported by a nebivolol mechanistic study performed in rat afferent arterioles [40]. Furthermore, Appel et al. reported pGC-A/cGMP pathway decreased mesangial cells contraction stimulated by Angiotensin II (ANG II) [41]. This supports that the GFR response to pGC-A/cGMP signaling also involves a reduction in mesangial cells contraction. Consistent with the data by Appel et al., an additional study reported that ANP dose dependently reduced the Ca^2+^ levels and glomerular mesangial cells contractility stimulated by arginine vasopressin [42]. In these latter experiments, similar results were obtained from exogenous administration of the cGMP analog 8-Br-cGMP [42].

## 4. Particulate GC-A/cGMP and Renal Protection

Apart from the acute physiological responses observed with natriuresis, diuresis and the increase in GFR, pGC-A/cGMP also actively contributes to long-term renal protection, including anti-fibrotic, anti-inflammatory, anti-apoptotic, and anti-renin-angiotensin-aldosterone (RAAS) properties. Direct evidence from pGC-A receptor, ANP and BNP genetically modified rodent models support that NP/pGC-A/cGMP plays a critical role in maintaining kidney physiological integrity and protecting the kidney from injury. Firstly, pGC-A gene disruption in rodents leads to kidney fibrosis and renal dysfunction [43,44]. Specifically, pro-inflammatory and pro-fibrotic proteins such as interleukin-6 (IL-6), interleukin-10 (IL-10), tumor necrosis factor alpha (TNF-α), Col-1, and TGF-β were upregulated in the kidney of pGC-A knockout mice compared to wild type mice, while overexpression of pGC-A reduced renal fibrosis, injury and downregulated deleterious proteins [44]. In addition, BNP gene NPPB knockout rats presented with renal interstitial fibrosis compared to wild type rats [45], with upregulation of fibrosis genes Col-1, TGF-β [45]. Furthermore, these genetically modified rats exhibited kidney injury such as tubular microcysts and glomerular sclerosis [45]. In contrast, ANP gene NPPA overexpression with adenovirus alleviated renal injury, including tubular damage, glomeruli sclerosis, and inflammatory cells infiltration in Dahl salt-sensitive rats [46].

Experiments also showed that cGMP attenuated AKI by enhancing mitochondria biogenesis and promoting mitochondria biogenesis related genes peroxisome proliferator-activated receptor gamma coactivator 1-alpha (PGC-1α), NADH dehydrogenase 1 beta subcomplex subunit 8 (NDUFβ8) in renal cortex and in renal proximal tubular cells [47]. This supports the concept that cGMP may protect the kidney through mitochondria regulation. Suppressing RAAS is another component of pGC-A/cGMP renal protective actions. Reduction of renin release by pGC-A/cGMP was highlighted by the use of ANP infusion in normal canines and cGMP analog 8-pCPT-cGMP treatment in renal juxtaglomerular cells [12,48]. Evidence of inhibiting ANG II mediated hypertrophic responses by ANP and 8-Br-cGMP in LLC-PK1 cells were highlighted by Hannken et al. These investigations proved that the inhibitory effect of ANP on ANG II induced cell hypertrophy was through antagonizing pro-hypertrophy molecule mitogen-activated protein kinase (MAPK) phosphorylation [49]. Results from the study by Pandey et al. were consistent with the hypertrophy study, using pGC-A receptor antagonist A71915 and cells overexpressing pGC-A [50]. In their study, ANP inhibited ANG II mediated MAPK activity, and the inhibition was abolished by pGC-A antagonist A71915. In contrast, pGC-A overexpression further enhanced the MAPK inhibitory effect compared to wild type cells.

Again PKG, the downstream target of pGC-A/cGMP, is involved in renal protection. Genetic deletion of PKG in mice resulted in enhanced renal fibrosis stimulated by unilateral ureter obstruction. After PKG was deleted, the inhibitory effects on pro-fibrotic genes TGF-β and fibronectin by cGMP were abolished [51], which highlights PKG’s protective role in anti-fibrosis through antagonizing TGF-β and fibronectin. Conversely, another study showed that PKG overexpression attenuated ischemia/reperfusion AKI in mice through a reduction in apoptosis mediated by a decline in Caspase 3 activity and Bcl-2, Bag-1 upregulation, and anti-inflammatory response secondary to inhibiting macrophage infiltration and cytokines such as interleukin 1β (IL-1β), TNF-α and IL-6 [52]. The protective role of PKG in the kidney was further supported by a study investigating cisplatin induced kidney injury in mice. Specifically, Maimaitiyiming et al. discovered that cisplatin treatment reduced PKG protein levels and activity [53] while PKG overexpression reduced renal apoptosis and pro-apoptotic genes such as Bax. Importantly, PKG overexpression reduced plasma creatinine and tubular damage in the mice. These findings clearly support the concept that PKG protects the kidney, and the renal protective effects are mediated through anti-fibrotic, anti-inflammatory, and anti-apoptotic properties. In addition to the abovementioned pathways, PKG was also demonstrated to protect the kidney through RAAS inhibition. Specifically, Gambaryan et al. demonstrated that the reduction in renin release in rat glomeruli by 8-pCPT-cGMP was reversed by the PKG inhibitor Rp-8-pCPT-cGMP. They further conducted PKG overexpression experiments and showed PKG overexpression enhanced the inhibitory effects on renin release [48] compared to wild type renal juxtaglomerular cells.

## 5. Novel Designer pGC-A Activator CRRL269

The numerous cellular, molecular, physiologic and pharmacological studies reviewed above strongly support pGC-A activators as potentially renal protective therapies. This is further supported by clinical trials as reported by Metzer and others [9,10]. While efficacy has been demonstrated by the use of ANP and BNP, advances in peptide engineering have resulted in the design of novel pGC activators [54]. This led to novel designer pGC activators that possess actions which go beyond the native activators. Such designer activators contain unique amino acid (AA) sequences that provide attractive biological properties making them potential innovative peptide therapeutics. Designer pGC activators transcend structural, biological, functional, and pharmacological properties of endogenous pGC activators. Hence designer activators retain the biological effects possessed by native activators such as natriuretic, diuretic and anti-RAAS properties. More importantly, however, peptide engineering may result in the development of novel peptides with enhanced activity or organ-selective actions which are not properties of native pGC-A activators. Specifically, potency of an activator may be enhanced due to greater resistance to the key enzyme neprilysin (NEP) degradation [55] or greater enhanced activator/receptor binding affinity.

**Figure 2:**
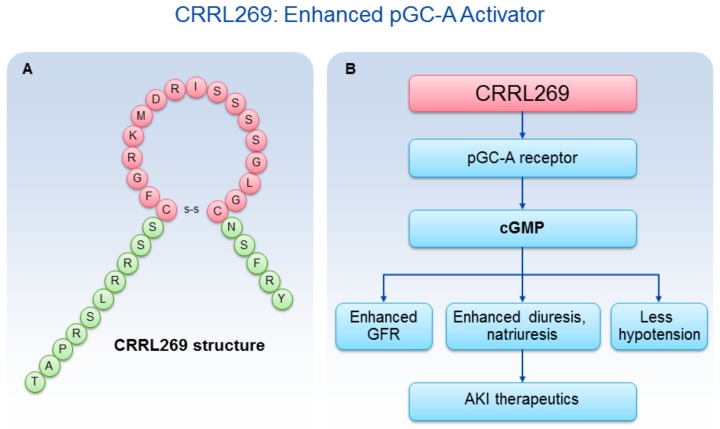
CRRL269 as an enhanced pGC-A activator. (**A**) CRRL269 structure; (**B**) CRRL269 implicated as a potential drug for AKI due to GFR increase, diuresis, natriuresis, and less hypotension.

**Figure 3:**
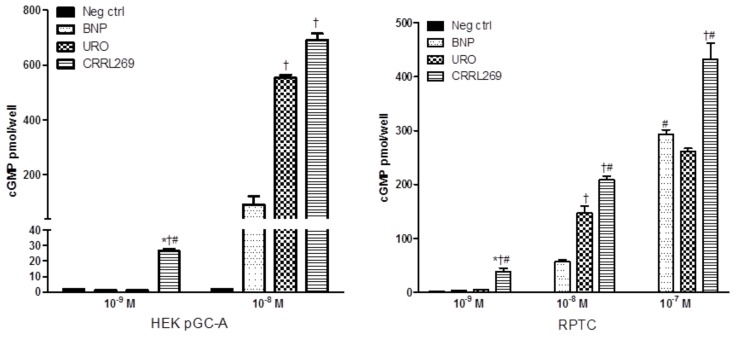
CRRL269 possesses enhanced pGC-A activator in vitro in human renal cells. CRRL269 generated significantly higher cGMP in HEK293 cells overexpressing pGC-A receptors (HEK pGC-A) and human renal proximal tubular cells (RPTC) compared to BNP or URO. Cells were treated with Hank’s balanced salt solution (HBSS, as negative control, neg ctrl), BNP, URO or CRRL269 for 10 min and cell lysates were collected for cGMP radioimmunoassay. * *p* < 0.05, versus neg ctrl, ^†^ *p* < 0.05, versus BNP, ^#^ *p* < 0.05, versus URO. American Journal of Physiology-Regulatory, Integrative and Comparative Physiology [27].

**Figure 4:**
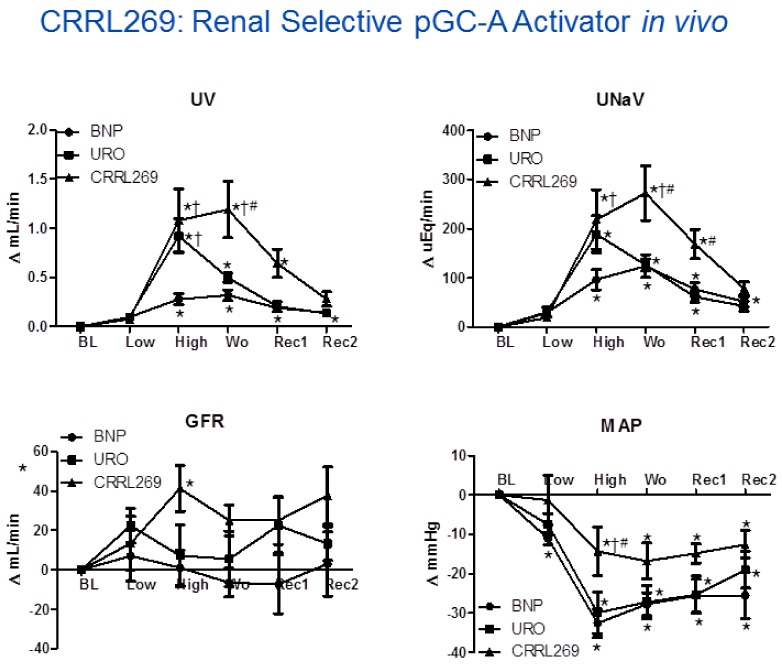
CRRL269 as an enhanced pGC-A activator in vivo in normal canines (*n* = 5). CRRL269 induced significantly higher and sustained diuresis (urine output, UV), natriuresis (urinary sodium excretion, UNaV), GFR, and lower blood pressure (mean arterial pressure, MAP) compared to BNP or URO. Acute studies were performed with intravenous infusion of low dose 2 pmol/kg/min and high dose 33 pmol/kg/min BNP, URO or CRRL269 in normal canines. Data are calculated from the difference from baseline. BL = baseline; Low = infusion of low dose 2 pmoL/kg/min BNP, URO or CRRL269; High = infusion of high dose 33 pmoL/kg/min; Wo = washout (0–30 min post-infusion); Rec1 = recovery 1, 30–60 min post-infusion; Rec2 = recovery 2, 60–90 min post-infusion. * *p* < 0.05, versus baseline (1-way ANOVA and Dunnett post-tests), ^†^ *p* < 0.05, versus BNP, ^#^ *p* < 0.05, versus URO (2-way ANOVA and Bonferroni post-hoc tests). American Journal of Physiology-Regulatory, Integrative and Comparative Physiology [27].

A goal from a renal protective perspective would be to develop a pGC-A activator with GFR enhancing, improved diuretic and natriuretic actions which could suppress the RAAS. Optimally, such activators would also possess less hypotensive properties, which has limited the therapeutic use of NPs such as BNP (i.e., nesiritide). Based on selective AA cassettes of BNP and URO, we designed a novel pGC-A activator, CRRL269 that possesses renal-selective properties [27] (Figure 2). In vitro experiments demonstrated that CRRL269 generated enhanced cGMP in HEK293 cells overexpressing pGC-A receptors and in primary human renal proximal tubular cells compared to native BNP and URO (Figure 3). This supports that CRRL269 manifests enhanced pGC-A receptor activity in renal cells. More importantly, in vivo experiments in normal canines demonstrated that CRRL269 induced enhanced diuresis, natriuresis and increased GFR with less reduction in blood pressure in comparison with native pGC-A activators BNP and URO (Figure 4). Mechanistic studies in vitro and ex vivo support that it has greater resistance to NEP degradation and reduced arterial relaxation compared to BNP and URO, which provides direct evidence for enhanced renal actions and reduced hypotension observed in vivo by CRRL269.

As stated above, naturally occurring pGC-A activators such as ANP, BNP or URO, inevitably reduce blood pressure which can decrease renal perfusion pressure and impair renal function while the renal actions may be further improved based upon rational drug design. Our recent pGC-A activator CRRL269, induced less vasorelaxation and less hypotensive effects. Furthermore, its renal actions including diuresis, natriuresis, and increase in GFR are more potent than native pGC-A activators. In addition, CRRL269 retains anti-RAAS action. These properties support that CRRL269 as a next generation renal selective pGC-A activator.

The enhanced renal actions observed with CRRL269 in normal canines support its potential clinical development in renal diseases such as AKI. As mentioned above, AKI patients manifest blunted renal function including GFR decline, reduced diuresis and natriuresis and novel effective drugs are an unmet need. Particulate GC-A/cGMP is a critical regulator of kidney function, which supports the development of novel pGC-A/cGMP activators with enhanced renal actions without hypotensive properties. In our recent study, CRRL269 does not induce hypotension as observed with other native pGC-A activators BNP and URO. This advantage adds to the efficacy and safety of CRRL269 for the prevention and treatment of AKI. Our future goals are to understand the protective roles of the CRRL269/cGMP pathway in renal cells and to further investigate CRRL269 therapeutic effects in an AKI model, relevant to clinical AKI pathophysiology in humans. With the exciting results published with recombinant ANP and BNP in AKI clinical trials [9,10], we see CRRL269 as a promising next generation pGC-A activator for AKI therapy.

In addition to AKI, the enhanced renal selective properties observed in vitro and in vivo may also support CRRL269 for the treatment of acute HF (AHF) and anti-remodeling of the heart and kidney. Diuretics such as furosemide are a mainstay therapy for AHF patients in the clinic due to its powerful effects to remove fluid retention in the body. However, furosemide activates RAAS and reduces GFR, which are associated with worse outcomes and prognosis in AHF patients [56]. As described above, CRRL269 stimulated GFR increase and suppressed RAAS in animal studies. Furthermore, previously in our laboratory, we demonstrated that BNP infusion in combination with furosemide significantly antagonized the RAAS activating effects induced by furosemide in an experiment HF canine model, compared to furosemide alone [57]. Thus, CRRL269 may represent a novel drug for AHF management. Additionally, the pluripotent actions of pGC-A/cGMP pathway such as anti-fibrotic, anti-inflammatory, and anti-apoptotic effects support CRRL269′s role in the long-term organ protection in cardiac and renal diseases.

In conclusion, a novel renal selective pGC-A activator, CRRL269 generates enhanced and renal specific cGMP activity in vitro and more importantly exerts potent GFR enhancing, less hypotensive and anti-RAAS properties. The pluripotent actions of CRRL269 support its potential development for AKI and other renal, cardiovascular diseases.

## Abbreviations

pGC-A	Particulate guanylyl cyclase A
NP	Natriuretic peptide
cGMP	3′,5′-Cyclic guanosine monophosphate
PKG	Protein kinase G
AKI	Acute kidney injury
GFR	Glomerular filtration rate
ANP	Atrial natriuretic peptide
BNP	B-type natriuretic peptide
URO	Urodilatin
PDE	Phosphodiesterase
NKA	Na^+^-K^+^-ATPase
IMCD	Inner medullary collect duct
VSMC	Vascular smooth muscle cell
HF	Heart failure
RAAS	Renin-angiotensin-aldosterone-system

## References

[1] Ogawa H., Qiu Y., Ogata C.M., Misono K.S. (2004). Crystal structure of hormone-bound atrial natriuretic peptide receptor extracellular domain: Rotation mechanism for transmembrane signal transduction. J. Biol. Chem..

[2] Lee C.Y., Burnett J.C. (2007). Natriuretic peptides and therapeutic applications. Heart Fail. Rev..

[3] Kellum J.A., Lameire N., Aspelin P., Barsoum R.S., Burdmann E.A., Goldstein S.L., Herzog C.A., Joannidis M., Kribben A., Levey A.S. (2012). Kidney disease: Improving global outcomes (KDIGO) acute kidney injury work group. KDIGO clinical practice guideline for acute kidney injury. Kidney Int. Suppl..

[4] Wonnacott A., Meran S., Amphlett B., Talabani B., Phillips A. (2014). Epidemiology and outcomes in community-acquired versus hospital-acquired AKI. Clin. J. Am. Soc. Nephrol..

[5] Hoste E.A., Bagshaw S.M., Bellomo R., Cely C.M., Colman R., Cruz D.N., Edipidis K., Forni L.G., Gomersall C.D., Govil D. (2015). Epidemiology of acute kidney injury in critically ill patients: The multinational AKI-EPI study. Intensive Care Med..

[6] Coca S.G., Singanamala S., Parikh C.R. (2012). Chronic kidney disease after acute kidney injury: A systematic review and meta-analysis. Kidney Int..

[7] Bansal N., Matheny M.E., Greevy R.A., Eden S.K., Perkins A.M., Parr S.K., Fly J., Abdel-Kader K., Himmelfarb J., Hung A.M. (2018). Acute Kidney Injury and Risk of Incident Heart Failure Among US Veterans. Am. J. Kidney Dis..

[8] De Caestecker M., Humphreys B.D., Liu K.D., Fissell W.H., Cerda J., Nolin T.D., Askenazi D., Mour G., Harrell F.E., Pullen N. (2015). Bridging Translation by Improving Preclinical Study Design in AKI. J. Am. Soc. Nephrol..

[9] Mentzer R.M., Oz M.C., Sladen R.N., Graeve A.H., Hebeler R.F., Luber J.M., Smedira N.G. (2007). Effects of perioperative nesiritide in patients with left ventricular dysfunction undergoing cardiac surgery: The NAPA Trial. J. Am. Coll. Cardiol..

[10] Sezai A., Hata M., Niino T., Yoshitake I., Unosawa S., Wakui S., Kimura H., Shiono M., Takayama T., Hirayama A. (2011). Results of low-dose human atrial natriuretic peptide infusion in nondialysis patients with chronic kidney disease undergoing coronary artery bypass grafting: The NU-HIT (Nihon University working group study of low-dose HANP Infusion Therapy during cardiac surgery) trial for CKD. J. Am. Coll. Cardiol..

[11] De Bold A.J., Borenstein H.B., Veress A.T., Sonnenberg H. (1981). A rapid and potent natriuretic response to intravenous injection of atrial myocardial extract in rats. Life Sci..

[12] Burnett J.C., Granger J.P., Opgenorth T.J. (1984). Effects of synthetic atrial natriuretic factor on renal function and renin release. Am. J. Physiol..

[13] Weidmann P., Hasler L., Gnadinger M.P., Lang R.E., Uehlinger D.E., Shaw S., Rascher W., Reubi F.C. (1986). Blood levels and renal effects of atrial natriuretic peptide in normal man. J. Clin. Investig..

[14] Colucci W.S., Elkayam U., Horton D.P., Abraham W.T., Bourge R.C., Johnson A.D., Wagoner L.E., Givertz M.M., Liang C.S., Neibaur M. (2000). Intravenous nesiritide, a natriuretic peptide, in the treatment of decompensated congestive heart failure. Nesiritide Study Group. N. Engl. J. Med..

[15] Nonoguchi H., Sands J.M., Knepper M.A. (1988). Atrial natriuretic factor inhibits vasopressin-stimulated osmotic water permeability in rat inner medullary collecting duct. J. Clin. Investig..

[16] Wang W., Li C., Nejsum L.N., Li H., Kim S.W., Kwon T.-H., Jonassen T.E., Knepper M.A., Thomsen K., Frøkiær J. (2006). Biphasic effects of ANP infusion in conscious, euvolumic rats: Roles of AQP2 and ENaC trafficking. Am. J. Physiol.-Renal Physiol..

[17] Garvin J.L. (1992). ANF inhibits norepinephrine-stimulated fluid absorption in rat proximal straight tubules. Am. J. Physiol..

[18] Cantiello H.F., Ausiello D.A. (1986). Atrial natriuretic factor and cGMP inhibit amiloride-sensitive Na^+^ transport in the cultured renal epithelial cell line, LLC-PK1. Biochem. Biophys. Res. Commun..

[19] Light D.B., Schwiebert E.M., Karlson K.H., Stanton B.A. (1989). Atrial natriuretic peptide inhibits a cation channel in renal inner medullary collecting duct cells. Science.

[20] Zeidel M.L., Seifter J.L., Lear S., Brenner B.M., Silva P. (1986). Atrial peptides inhibit oxygen consumption in kidney medullary collecting duct cells. Am. J. Physiol..

[21] Zeidel M.L., Silva P., Brenner B.M., Seifter J.L. (1987). cGMP mediates effects of atrial peptides on medullary collecting duct cells. Am. J. Physiol..

[22] Scavone C., Scanlon C., McKee M., Nathanson J.A. (1995). Atrial natriuretic peptide modulates sodium and potassium-activated adenosine triphosphatase through a mechanism involving cyclic GMP and cyclic GMP-dependent protein kinase. J. Pharmacol. Exp. Ther..

[23] Light D.B., Corbin J.D., Stanton B.A. (1990). Dual ion-channel regulation by cyclic GMP and cyclic GMP-dependent protein kinase. Nature.

[24] Jin X.H., Siragy H.M., Carey R.M. (2001). Renal interstitial cGMP mediates natriuresis by direct tubule mechanism. Hypertension.

[25] MacFarland R.T., Zelus B.D., Beavo J.A. (1991). High concentrations of a cGMP-stimulated phosphodiesterase mediate ANP-induced decreases in cAMP and steroidogenesis in adrenal glomerulosa cells. J. Biol. Chem..

[26] Nikolaev V.O., Gambaryan S., Engelhardt S., Walter U., Lohse M.J. (2005). Real-time monitoring of the PDE2 activity of live cells: Hormone-stimulated cAMP hydrolysis is faster than hormone-stimulated cAMP synthesis. J. Biol. Chem..

[27] Chen Y., Harty G.J., Huntley B.K., Iyer S.R., Heublein D.M., Harders G.E., Meems L.M.G., Pan S., Sangaralingham S.J., Ichiki T. (2017). CRRL269: A Novel Designer and Renal Enhancing pGC-A Peptide Activator. Am. J. Physiol.-Regul Integr. Comp. Physiol..

[28] Holmes S.J., Espiner E.A., Richards A.M., Yandle T.G., Frampton C. (1993). Renal, endocrine, and hemodynamic effects of human brain natriuretic peptide in normal man. J. Clin. Endocrinol. Metab..

[29] Chen H.H., Huntley B.K., Schirger J.A., Cataliotti A., Burnett J.C. (2006). Maximizing the renal cyclic 3′-5′-guanosine monophosphate system with type V phosphodiesterase inhibition and exogenous natriuretic peptide: A novel strategy to improve renal function in experimental overt heart failure. J. Am. Soc. Nephrol..

[30] Huang C.L., Ives H.E., Cogan M.G. (1986). In vivo evidence that cGMP is the second messenger for atrial natriuretic factor. Proc. Natl. Acad. Sci. USA.

[31] Richards A.M., Tonolo G., Montorsi P., Finlayson J., Fraser R., Inglis G., Towrie A., Morton J.J. (1988). Low dose infusions of 26- and 28-amino acid human atrial natriuretic peptides in normal man. J. Clin. Endocrinol. Metab..

[32] Kwon O., Hong S.-M., Ramesh G. (2009). Diminished NO generation by injured endothelium and loss of macula densa nNOS may contribute to sustained acute kidney injury after ischemia-reperfusion. Am. J. Physiol.-Renal Physiol..

[33] Lee C.Y., Huntley B.K., McCormick D.J., Ichiki T., Sangaralingham S.J., Lisy O., Burnett J.C. (2015). Cenderitide: Structural requirements for the creation of a novel dual particulate guanylyl cyclase receptor agonist with renal-enhancing in vivo and ex vivo actions. Eur. Heart J.-Cardiovasc. Pharmacother..

[34] Kikkawa R., Haneda M., Sakamoto K., Koya D., Shikano T., Nakanishi S., Matsuda Y., Shigeta Y. (1993). Antagonist for atrial natriuretic peptide receptors ameliorates glomerular hyperfiltration in diabetic rats. Biochem. Biophys. Res. Commun..

[35] Rashatwar S.S., Cornwell T.L., Lincoln T.M. (1987). Effects of 8-bromo-cGMP on Ca^2+^ levels in vascular smooth muscle cells: Possible regulation of Ca^2+^-ATPase by cGMP-dependent protein kinase. Proc. Natl. Acad. Sci. USA.

[36] Kubo M., Nakaya Y., Matsuoka S., Saito K., Kuroda Y. (1994). Atrial natriuretic factor and isosorbide dinitrate modulate the gating of ATP-sensitive K^+^ channels in cultured vascular smooth muscle cells. Circ. Res..

[37] Burnett J.C., Opgenorth T.J., Granger J.P. (1986). The renal action of atrial natriuretic peptide during control of glomerular filtration. Kidney Int..

[38] Dunn B.R., Ichikawa I., Pfeffer J.M., Troy J.L., Brenner B.M. (1986). Renal and systemic hemodynamic effects of synthetic atrial natriuretic peptide in the anesthetized rat. Circ. Res..

[39] Marin-Grez M., Fleming J.T., Steinhausen M. (1986). Atrial natriuretic peptide causes pre-glomerular vasodilatation and post-glomerular vasoconstriction in rat kidney. Nature.

[40] Feng M.-G., Prieto M.C., Navar L.G. (2012). Nebivolol-induced vasodilation of renal afferent arterioles involves β3-adrenergic receptor and nitric oxide synthase activation. Am. J. Physiol.-Renal Physiol..

[41] Appel R.G., Wang J., Simonson M.S., Dunn M.J. (1986). A mechanism by which atrial natriuretic factor mediates its glomerular actions. Am. J. Physiol..

[42] Meyer-Lehnert H., Tsai P., Caramelo C., Schrier R.W. (1988). ANF inhibits vasopressin-induced Ca^2+^ mobilization and contraction in glomerular mesangial cells. Am. J. Physiol..

[43] Yoshihara F., Tokudome T., Kishimoto I., Otani K., Kuwabara A., Horio T., Kawano Y., Kangawa K. (2015). Aggravated renal tubular damage and interstitial fibrosis in mice lacking guanylyl cyclase-A (GC-A), a receptor for atrial and B-type natriuretic peptides. Clin. Exp. Nephrol..

[44] Kumar P., Gogulamudi V.R., Periasamy R., Raghavaraju G., Subramanian U., Pandey K.N. (2017). Inhibition of HDAC enhances STAT acetylation, blocks NF-kappaB, and suppresses the renal inflammation and fibrosis in Npr1 haplotype male mice. Am. J. Physiol.-Renal Physiol..

[45] Holditch S.J., Schreiber C.A., Nini R., Tonne J.M., Peng K.W., Geurts A., Jacob H.J., Burnett J.C., Cataliotti A., Ikeda Y. (2015). B-Type Natriuretic Peptide Deletion Leads to Progressive Hypertension, Associated Organ Damage, and Reduced Survival: Novel Model for Human Hypertension. Hypertension.

[46] Lin K.F., Chao J., Chao L. (1998). Atrial natriuretic peptide gene delivery attenuates hypertension, cardiac hypertrophy, and renal injury in salt-sensitive rats. Hum. Gene Ther..

[47] Whitaker R.M., Wills L.P., Stallons L.J., Schnellmann R.G. (2013). cGMP-selective phosphodiesterase inhibitors stimulate mitochondrial biogenesis and promote recovery from acute kidney injury. J. Pharmacol. Exp. Ther..

[48] Gambaryan S., Wagner C., Smolenski A., Walter U., Poller W., Haase W., Kurtz A., Lohmann S.M. (1998). Endogenous or overexpressed cGMP-dependent protein kinases inhibit cAMP-dependent renin release from rat isolated perfused kidney, microdissected glomeruli, and isolated juxtaglomerular cells. Proc. Natl. Acad. Sci. USA.

[49] Hannken T., Schroeder R., Stahl R.A., Wolf G. (2001). Atrial natriuretic peptide attenuates ANG II-induced hypertrophy of renal tubular cells. Am. J. Physiol.-Renal Physiol..

[50] Pandey K.N., Nguyen H.T., Li M., Boyle J.W. (2000). Natriuretic peptide receptor-A negatively regulates mitogen-activated protein kinase and proliferation of mesangial cells: Role of cGMP-dependent protein kinase. Biochem. Biophys. Res. Commun..

[51] Schinner E., Schramm A., Kees F., Hofmann F., Schlossmann J. (2013). The cyclic GMP-dependent protein kinase Ialpha suppresses kidney fibrosis. Kidney Int..

[52] Li Y., Tong X., Maimaitiyiming H., Clemons K., Cao J.M., Wang S. (2012). Overexpression of cGMP-dependent protein kinase I (PKG-I) attenuates ischemia-reperfusion-induced kidney injury. Am. J. Physiol.-Renal Physiol..

[53] Maimaitiyiming H., Li Y., Cui W., Tong X., Norman H., Qi X., Wang S. (2013). Increasing cGMP-dependent protein kinase I activity attenuates cisplatin-induced kidney injury through protection of mitochondria function. Am. J. Physiol.-Renal Physiol..

[54] Meems L.M.G., Burnett J.C. (2016). Innovative Therapeutics: Designer Natriuretic Peptides. JACC Basic Transl. Sci..

[55] Chen Y., Burnett J.C. (2017). Biochemistry, Therapeutics, and Biomarker Implications of Neprilysin in Cardiorenal Disease. Clin. Chem..

[56] Felker G.M., O’Connor C.M., Braunwald E. (2009). Loop diuretics in acute decompensated heart failure: Necessary? Evil? A necessary evil?. Circ. Heart Fail..

[57] Cataliotti A., Boerrigter G., Costello-Boerrigter L.C., Schirger J.A., Tsuruda T., Heublein D.M., Chen H.H., Malatino L.S., Burnett J.C. (2004). Brain natriuretic peptide enhances renal actions of furosemide and suppresses furosemide-induced aldosterone activation in experimental heart failure. Circulation.

